# Toward optimal inline respiratory motion correction for in vivo cardiac diffusion tensor MRI using symmetric and inverse‐consistent deformable image registration

**DOI:** 10.1002/mrm.30485

**Published:** 2025-03-10

**Authors:** Yuchi Liu, Danielle Kara, Thomas Garrett, Shi Chen, Daniel Wee, Ning Jin, Peter Speier, Hiroshi Nakagawa, Pasquale Santangeli, Michael A. Bolen, Oussama Wazni, Mazen Hanna, W. H. Wilson Tang, Animesh Tandon, Deborah Kwon, Xiaoming Bi, Christopher Nguyen

**Affiliations:** ^1^ Cardiovascular MR R&D Collaborations Siemens Medical Solutions USA, Inc. Cleveland Ohio USA; ^2^ Cardiovascular Innovation Research Center Heart Vascular Thoracic Institute, Cleveland Clinic Cleveland Ohio USA; ^3^ Department of Diagnostic Radiology Imaging Institute, Cleveland Clinic Cleveland Ohio USA; ^4^ Cardiovascular MR R&D Collaborations Siemens Medical Solutions USA, Inc. Columbus Ohio USA; ^5^ Siemens Healthineers AG Erlangen Germany; ^6^ Cardiovascular Medicine Heart Vascular Thoracic Institute, Cleveland Clinic Cleveland Ohio USA; ^7^ Department of Biomedical Engineering Case Western Reserve University and Cleveland Clinic Cleveland Ohio USA; ^8^ Department of Heart, Vascular, and Thoracic Children's Institute, Cleveland Clinic Cleveland Ohio USA; ^9^ Cardiovascular MR R&D Collaborations Siemens Medical Solutions USA, Inc. Chicago Illinois USA

**Keywords:** cardiac magnetic resonance, diffusion tensor imaging, motion correction

## Abstract

**Purpose:**

This study aims to develop a free‐breathing cardiac DTI method with fast and robust motion correction.

**Methods:**

Two proposed image registration‐based motion correction (MOCO) strategies, MOCO_Naive_ and MOCO_Avg_, were applied to diffusion‐weighted images acquired with M2 diffusion gradients under free‐breathing. The effectiveness of MOCO was assessed by tracking epicardium pixel positions across image frames. Resulting mean diffusivity (MD), fractional anisotropy (FA), and helix angle (HA) maps were compared against a previous low rank tensor based MOCO method (MOCO_LRT_) in 20 healthy volunteers and two patients scanned at 3 T.

**Results:**

Compared with the MOCO_LRT_ method, both proposed MOCO_Naive_ and MOCO_Avg_ methods generated slightly lower MD and helix angle transmurality (HAT) magnitude values, and significantly lower FA values. Moreover, both proposed MOCO methods achieved significantly smaller SDs of MD and FA values, and more smoothly varying helical structure in HA maps in healthy volunteers, indicating more effective MOCO. Elevated MD, decreased FA, and lower HAT magnitude were observed in two patients compared with healthy volunteers. Furthermore, the computing speed of image registration‐based MOCO is twice as fast as the LRT method on the same dataset and same workstation.

**Conclusion:**

This study demonstrates a fast and robust motion correction approach using image registration for in vivo free‐breathing cardiac DTI. It improves the quality of quantitative diffusion maps and will facilitate clinical translation of cardiac DTI.

## 1 INTRODUCTION

Cardiac DTI provides a non‐invasive tool based on measurements of MR signals sensitive to local diffusion of water molecules that reflect tissue structure. DTI has shown promise in depiction of microstructure in the heart,^1^, ^2^ as well as alterations in cardiac diseases.^3^, ^4^, ^5^ A recent study also shows that changes in myocardial microstructure as measured by cardiac DTI are promising early‐phenotype biomarkers.^6^ However, cardiac DTI is challenging for multiple reasons. First, SNR of the acquired diffusion‐weighted images is relatively low because of diffusion encoding and long echo‐time (TE). Second, bulk motion induced signal dropout requires DTI methods to minimize the effects of cardiac motion and respiratory motion during data acquisition. This can be achieved by shortening the duration of diffusion encoding and/or developing diffusion gradients relatively insensitive to bulk motion. Third, long acquisition time requires either repeated breath‐holds or motion correction (MOCO) under free‐breathing to co‐register diffusion‐weighted images acquired from different heart beats at different respiratory states.

To ameliorate motion effects, cardiac DTI techniques are categorized into stimulated echo acquisition mode (STEAM) and spin‐echo (SE) based acquisitions. Although both approaches use single‐shot EPI with electrocardiography (ECG) triggering, STEAM acquisitions require multiple breath‐holds and are vulnerable to strain contamination; the SE‐based method with first and second order motion compensated (M2) diffusion gradients enable robust cardiac DTI with free‐breathing.^7^ A low‐rank tensor (LRT) model has been proposed to correct respiratory motion for the SE‐based free‐breathing M2 cardiac DTI method by regressing out *b*‐value contrast before image registration.^8^ LRT decomposition is applied to diffusion‐weighted images, isolating diffusion contrast to create respiratory bin templates. The transforms that co‐register these bin templates are applied to original diffusion‐weighted images to produce motion corrected images. Recently deep learning techniques have also been used for image registration and segmentation in cardiac DTI.^9^

Alternatively, an image registration method^10^ has been successfully applied for MOCO to a wide range of cardiovascular magnetic resonance techniques such as perfusion, T_1_ mapping, free‐breathing phase sensitive inversion recovery (PSIR), etc.^11^, ^12^, ^13^ T_1_ mapping tackles the challenge of contrast change by iterative synthetic contrast estimate, whereas in perfusion applications each image is registered to the adjacent one with similar contrast. The idea of direct image registration of diffusion‐weighted images has been explored in a previous study.^14^ However, the limitations of SNR and diffusion contrast still presents challenges in generating high‐quality maps.

This study aims to apply the image registration‐based MOCO method described above to free‐breathing M2 SE‐based cardiac DTI data acquired on a 3 T MRI scanner. Two MOCO strategies were proposed and compared against the previously proposed LRT MOCO method in healthy volunteers and cardiac patients.

## 2 METHODS

### 2.1 Human data acquisition

A research sequence using M2 diffusion gradients^15^ and EPI readout was performed in 20 healthy volunteers (12 female, 20.7 ± 8.1 years old) and two patients (patient 1 is male, 66 years old, with non‐ischemic cardiomyopathy; patient 2 is male, 63 years old, with suspicious sarcoid) under an institutional review board approved protocol. All DTI experiments were performed on a 3 T MRI scanner (MAGNETOM Cima. X, Siemens Healthineers, Forchheim, Germany) capable of maximum gradient strength 200 mT/m and maximum slew rate 200 mT/m/s. The actual maximum gradient amplitude on a single gradient axis used in the sequence was 120 mT/m/s because of the design of the asymmetric trapezoidal diffusion gradient waveform and diffusion encoding parameters. Cardiac DTI scans used FOV 350 mm × 131 mm; matrix size 128 × 48; inner volume excitation^16^, ^17^, ^18^; in‐plane resolution 2.7 mm × 2.7 mm; 5 slices with 8 mm thickness; TR of 5 R‐R intervals; 12 diffusion directions; 1 average for b0 (*b* = 50 s/mm^2^) followed by 8 averages for high *b* value (*b* = 500 s/mm^2^). TE was 59 ms without exceeding peripheral nerve stimulation limit. All images were acquired at end systole with ECG triggering under free‐breathing. As a result of difference in subjects' heart rate, the total cardiac DTI acquisition time for all five slices was 8 min 47 s ± 1 min 27 s in volunteers, 9 min 52 s in patient 1, and 13 min 25 s in patient 2.

### 2.2 MOCO strategies

Two proposed MOCO strategies using pair‐wise symmetric and inverse‐consistent deformable (non‐rigid) image registration^10^ were performed directly on acquired diffusion‐weighted images for all human data without probing diffusion contrast. Note that this registration method is a different algorithm compared to the “conventional MOCO” reported in a previous study,^8^ which was performed using an open‐source software^19^ and was shown to be less effective than LRT MOCO. The LRT based multitasking MOCO approach was also performed in this study as a comparison.^8^ The first method (MOCO_Naive_) naively registered all individual diffusion‐weighted images to the first acquired b50 image (Figure S1A). The second method (MOCO_Avg_) registered all 12 b50 images to the first acquired b50 image to create an averaged reference. The average of the 12 b50 images after MOCO was used as the reference image for the following steps because of its relatively high SNR. Individual high *b*‐value images were registered to the above reference image followed by averaging for each diffusion direction. Averaged high *b*‐value images were registered to the reference image again (Figure S1B). Both proposed MOCO strategies were performed in MATLAB (The MathWorks, 2023a).

To demonstrate the effectiveness of the proposed MOCO strategies, a line across the heart from head to foot direction (i.e., from anterior toward inferior in short‐axis view) over all diffusion‐weighted image frames after interpolation was plotted for original free‐breathing images, as well as images resulting from MOCO_LRT_, MOCO_Naive_, and MOCO_Avg_, respectively. The position of the epicardium pixel over image frames was captured by calculating the first‐order derivative of the line plot and picking up the index of the pixel corresponding to the largest first‐order derivative value in a 10‐pixel range around epicardium along the line. The line was placed manually and derivative calculation did not involve filters. SD of the epicardium pixel indices over all image frames was calculated to indicate the effectiveness of different MOCO strategies. A Student *t* test was performed to compare the SDs resulting from different MOCO methods for each slice as well as global, which was calculated as the average of all five slices. Statistical significance was accepted at *p* < 0.05 and Bonferroni correction was performed for multiple comparisons.

### 2.3 DTI fitting and data analysis

After MOCO using proposed MOCO_Naive_ and MOCO_Avg_ methods as well as the previous MOCO_LRT_ method described above, mean diffusivity (MD), fractional anisotropy (FA), and helix angle (HA) maps were generated for each slice using a custom built, Python‐based DTI processing library. Image interpolation was performed before tensor fitting to achieve effective spatial resolution of 1.4 mm × 1.4 mm in the resulting diffusion maps. Mean and SD of MD, FA, and helix angle transmurality (HAT) maps for each slice as well as for global (all five slices) were calculated by manually segmenting the left ventricle (LV). Diffusion parameter maps were similarly generated using original free‐breathing data without MOCO. Resulting diffusion parameter values from MOCO_Naive_, MOCO_Avg_, MOCO_LRT_, and free‐breathing data were compared using a Student *t* test. Statistical significance was accepted at *p* < 0.05 and Bonferroni correction was performed for multiple comparisons.

## 3 RESULTS

**FIGURE 1:**
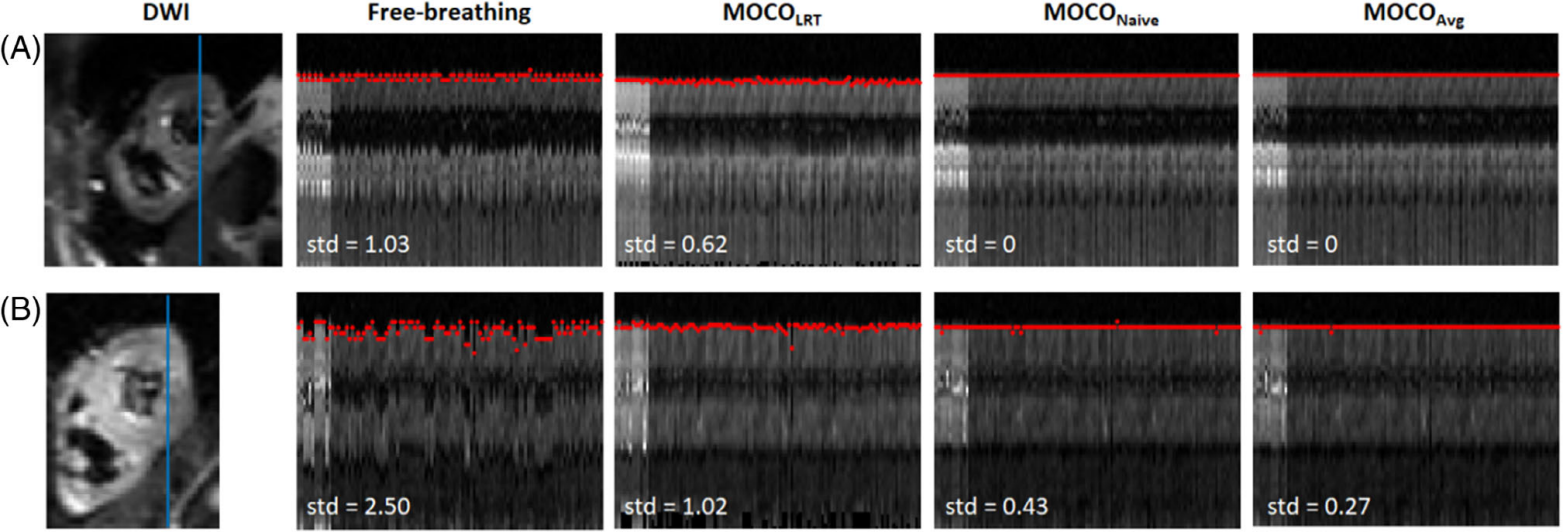
A line across the heart (indicated by the blue line in the DWI image) in two healthy volunteers is plotted across all frames for free‐breathing data, and images resulting from motion correction (MOCO) methods MOCO_LRT_, MOCO_Naive_, and MOCO_Avg_. Epicardium pixels are labeled with red circles. SD of the epicardium pixel positions across all image frames are also labeled. (A) shows a case with less motion before MOCO compared with (B).

Both proposed MOCO methods took approximately 47 s for a dataset with five slices and 540 total images on a workstation equipped with 32GB RAM and 11th i7 2.5GHz processor. In contrast, the computing time doubled (i.e., 90s) for the previously proposed MOCO_LRT_ method^8^ for the same dataset and same workstation. Figure 1 shows respiratory motion across image frames in the original free‐breathing images and the motion corrected images in two healthy volunteers by plotting a line in head‐to‐foot direction across the heart over all frames (the case shown in Figure 1A has less motion before MOCO). Epicardium positions were successfully detected in both free‐breathing images and images after MOCO (labeled in red circles). SDs of the epicardium positions across all image frames are also shown for each MOCO method. SDs of the epicardium pixel positions across all image frames in all volunteers are summarized in Table S1. For all slices, the proposed two MOCO methods achieved significantly smaller SDs compared with MOCO_LRT_ method (*p* < 0.005), and all three MOCO methods achieved significantly smaller SDs compared with free‐breathing (*p* < 0.001). No significant difference was observed between the two proposed MOCO methods. Moreover, all three MOCO methods achieved better MOCO as indicated by smaller SDs in midventricular slices compared with basal and apical slices, which are more prone to through‐plane motion.

**FIGURE 2:**
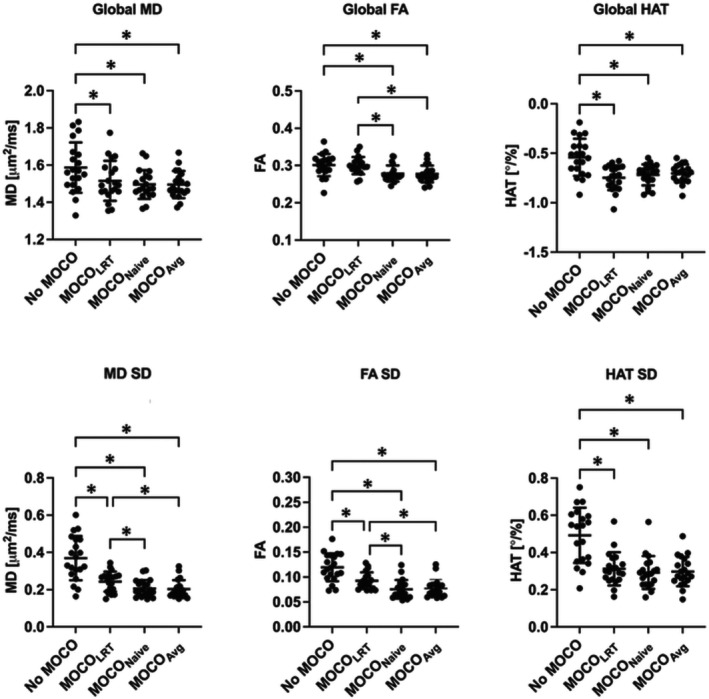
Average and SD of the global mean diffusivity (MD), fractional anisotropy (FA), and helix angle transmurality (HAT) values in all volunteers resulting from (1) free‐breathing without motion correction (MOCO); (2) MOCO_LRT_; (3) MOCO_Naive_; (4) MOCO_Avg_. Statistically significant difference (*p* < 0.05) is indicated by asterisk.

Figure 2 shows average and SD of the global MD, FA, and HAT values in all volunteers. Compared with MOCO_LRT_ (MD, 1.52 ± 0.10 μm^2^/ms; FA, 0.30 ± 0.02; HAT, −0.75°/% ± 0.12°/%), proposed MOCO_Naive_ strategy yielded slightly lower MD (1.50 ± 0.08 μm^2^/ms), significantly lower FA (0.28 ± 0.02, *p* < 0.001), and slightly lower HAT magnitude values (−0.72°/% ± 0.10°/%); MOCO_Avg_ generated slightly lower MD (1.50 ± 0.07 μm^2^/ms), significantly lower FA (0.28 ± 0.02, *p* < 0.001), and lower HAT magnitude values (−0.70°/% ± 0.09°/%). SDs were significantly reduced in MD (*p* < 0.001) and FA (*p* < 0.001) and similar in HAT for both proposed MOCO strategies compared with MOCO_LRT_. All results generated by MOCO_Naive_ and MOCO_Avg_ were similar without statistically significant difference. In addition, the values generated from free‐breathing data without MOCO are significantly different compared with those after MOCO (*p* < 0.001) except for global FA values between no MOCO and MOCO_LRT_. Low image quality with severe heterogeneity in LV of the diffusion parameter maps generated without MOCO was observed and shown in a healthy volunteer in Figure S2.

**FIGURE 3:**
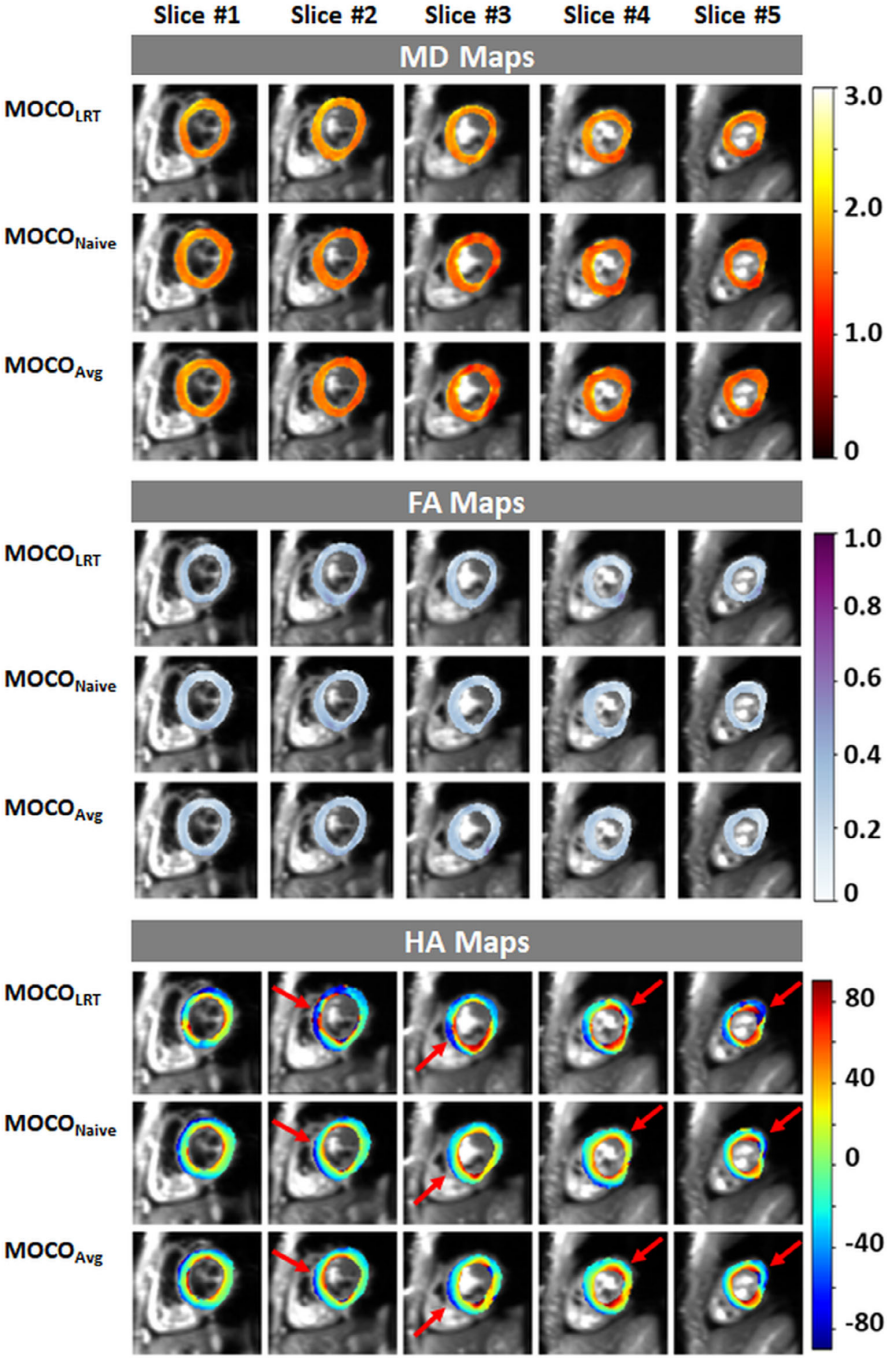
Mean diffusivity (MD), fractional anisotropy (FA), and helix angle (HA) maps of all five slices in a volunteer generated by motion correction (MOCO) method MOCO_LRT_, MOCO_Naive_, and MOCO_Avg_. MOCO_Naive_ and MOCO_Avg_ achieved overall more smoothly varied helical structure compared with MOCO_LRT_ (indicated by red arrows in HA maps).

MD, FA, and HA maps of all five slices in a volunteer are shown in Figure 3. All MOCO methods generated good image quality. However, HA maps generated by proposed MOCO methods achieved more smoothly varying helical structure (indicated by red arrows) because of more robust MOCO compared to the MOCO_LRT_ method.

**FIGURE 4:**
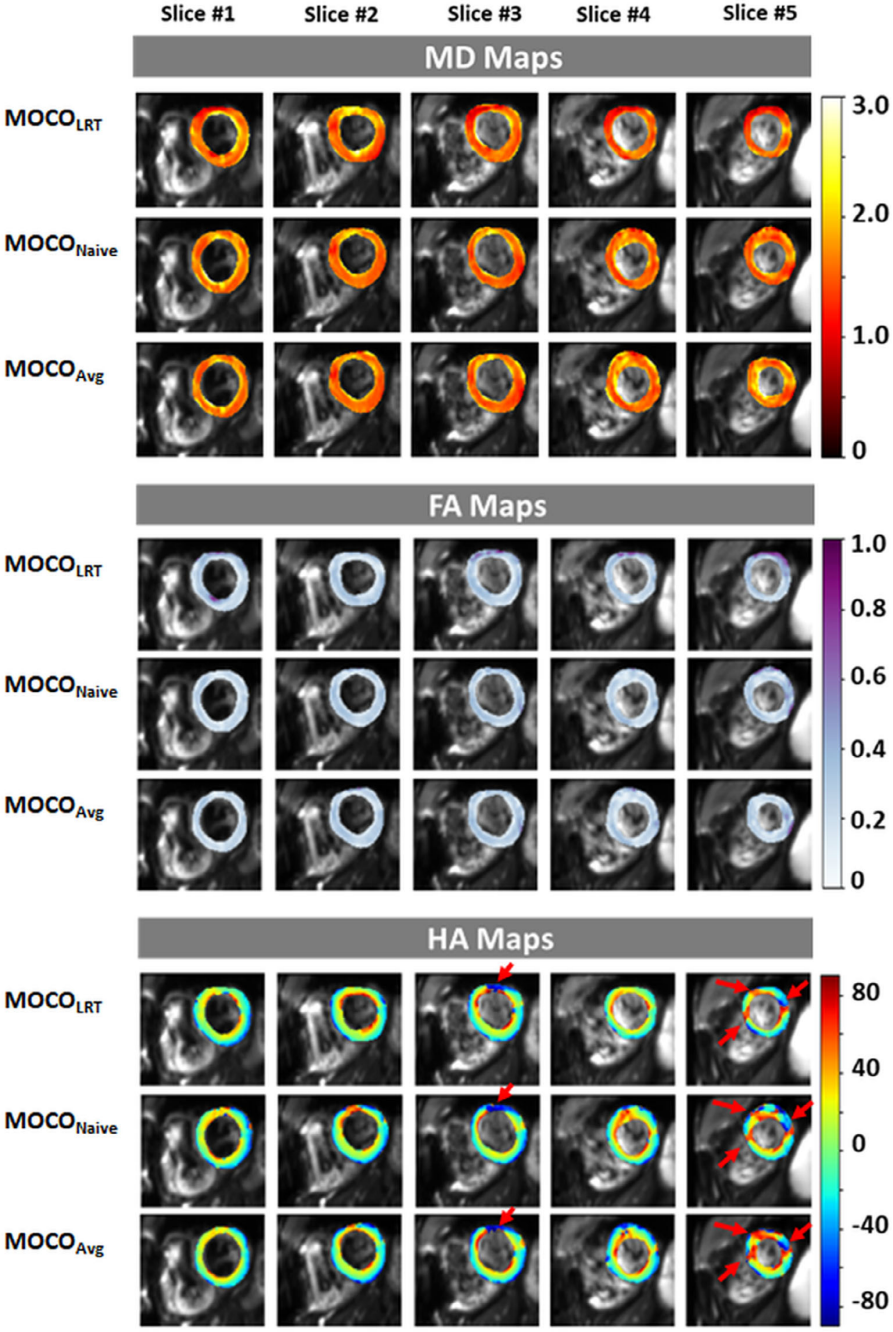
Mean diffusivity (MD), fractional anisotropy (FA), and helix angle (HA) maps of all five slices in patient 1 generated by motion correction (MOCO) method MOCO_LRT_, MOCO_Naive_, and MOCO_Avg_. Disruption of the helical structure in some regions is indicated by red arrows in HA maps.

**FIGURE 5:**
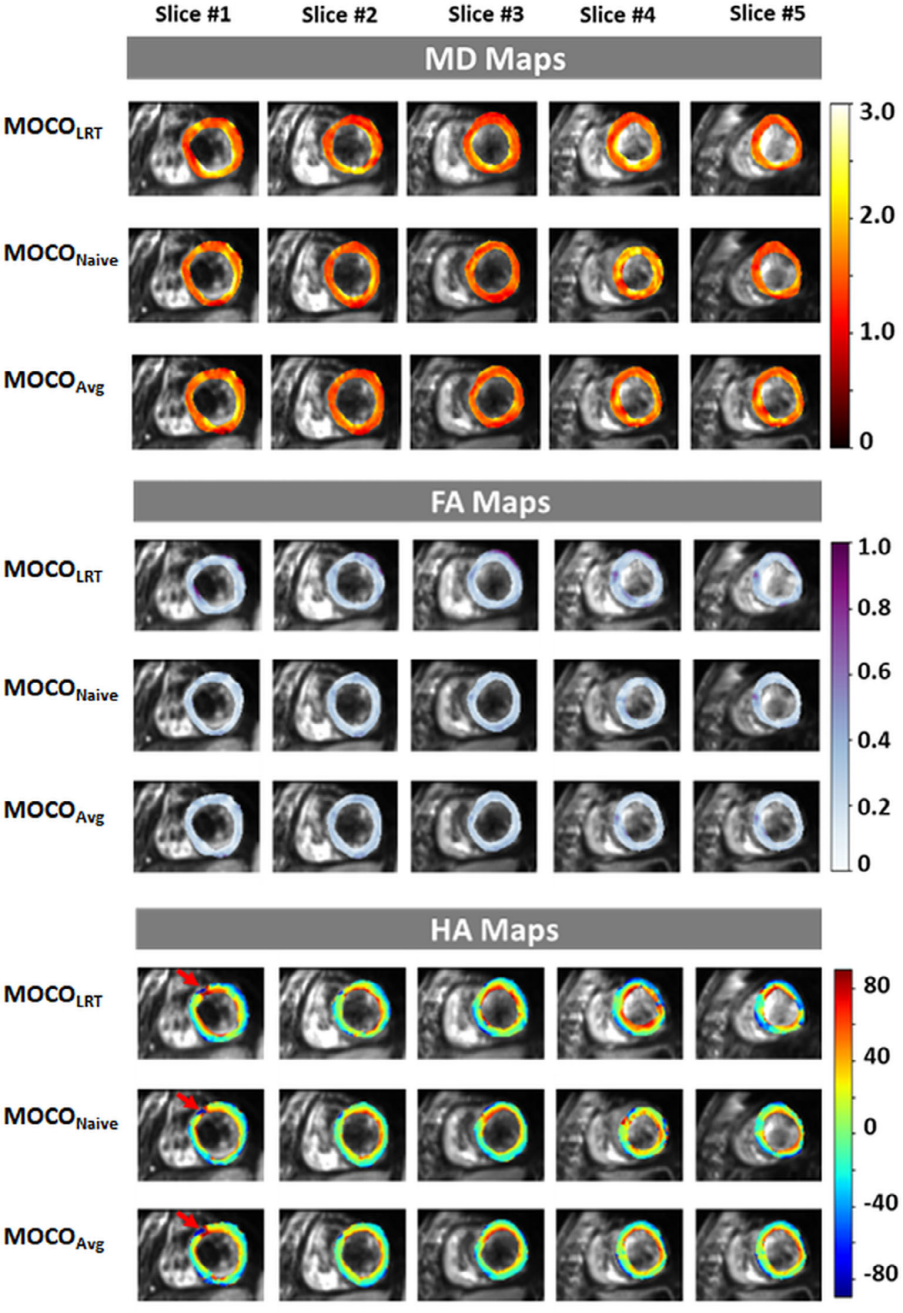
Mean diffusivity (MD), fractional anisotropy (FA), and helix angle (HA) maps of all five slices in patient 2 generated by motion correction (MOCO) method MOCO_LRT_, MOCO_Naive_, and MOCO_Avg_. Disruption of the helical structure in some regions is indicated by red arrows in HA maps.

Global average and SDs of MD, FA, HAT values in the two patients are summarized in Table S2. In patient 1 with non‐ischemic cardiomyopathy, elevated MD and lower FA were observed using proposed MOCO methods compared with MOCO_LRT_. Global HAT value was comparable between MOCO_Naive_ and MOCO_LRT_, lower magnitude in MOCO_Avg_ than MOCO_LRT_. Figure 4 shows MD, FA, and HA maps in all five slices in patient 1. Disruption of helical structure was observed in anterior and anterolateral regions in mid/apical slices, which correlated with late gadolinium enhancements (LGE) as shown in Figure S3. The HA maps also depicted less variation of helix angle from endocardium to epicardium compared with healthy volunteers. In patient 2 with suspicious sarcoid, lower global MD, comparable FA, and lower global HAT magnitude values were observed using proposed MOCO methods compared with MOCO_LRT_ (Table S2). Figure 5 shows MD, FA, and HA maps in all five slices in patient 2. Noticeable large variations in MD map of slice 4 resulting from MOCO_Naive_ was because of failed MOCO. Figure S4 shows individual DWI images resulting from proposed MOCO methods in all five slices in patient 2. Large deformation in the myocardium in slice 4 caused by failed MOCO from MOCO_Naive_ can be observed visually (indicated by red box). MD, FA, and HAT values of each individual slice are also shown in Table S3. Except for slice 4 resulting from MOCO_Naive_, proposed MOCO methods yield less variations in helical structure (i.e., smaller absolute HAT values) compared with healthy volunteers, indicating compromised cardiac tissue in consistent with late gadolinium enhancement shown in the LGE images (Figure S5). In anteroseptal wall of LV in the basal slice (Figure S5B), disruption of helical structure in the HA map was observed, verified by enhancement in the LGE image in the same area. This enhancement pattern in LGE images is consistent with previous reports on cardiac sarcoid cases.^20^, ^21^ In the lateral wall of LV, in the apical slice where LGE revealed infarction (Figure S5C), MOCO_LRT_ showed abrupt changes of helical structure, which is supported by some previous findings.^22^ Although the proposed MOCO_Naive_ and MOCO_Avg_ methods showed smoother transition of the fiber orientations from endocardium to epicardium, which is consistent with some previous reports that observed preservation of original fiber orientation in infarction.^23^, ^24^, ^25^

## 4 DISCUSSION

This study shows the feasibility and robustness of respiratory MOCO for in vivo free‐breathing cardiac DTI using pair‐wise symmetric and inverse‐consistent deformable image registration. Compared with the previous LRT‐based method, MOCO was performed directly on diffusion‐weighted images without the need to regress out diffusion contrast. This simplified process reduces computing time to half and is more practical to be implemented inline to generate cardiac diffusion results on the scanner. In volunteers, both proposed schemes are more robust than MOCO_LRT_ reflected by smaller variation of the epicardium pixel positions across all image frames, visually smoother helical structure in HA maps, as well as smaller variation in the MD and FA values. In a small number of patients, MOCO_Avg_ was found more robust than MOCO_Naive_. Among the 10 slices acquired in two patients, MOCO_Naive_ failed in one slice, whereas no apparent failure was observed for both MOCO_Avg_ and MOCO_LRT_.

Previous studies using SE based M2 diffusion sequences^7^, ^8^, ^26^, ^27^, ^28^, ^29^, ^30^ have reported a wide range of quantitative diffusion parameters at systole in healthy volunteers: global MD values from 1.26 to 1.72 μm^2^/ms; FA values from 0.28 to 0.37; HAT values from −0.8°/% to −1.1°/%. The difference in results is possibly associated with different acquisition parameters (e.g., *b* value, number of diffusion directions), image quality resulting from different TEs (61–76 ms) and acceleration methods (e.g., parallel imaging vs. inner volume excitation), and different MOCO/motion compensation methods. Global MD, FA, and HAT values resulting from all MOCO methods in volunteers observed in the current study (MD, 1.49–1.52 μm^2^/ms; FA, 0.29–0.31; HAT, −0.73°/% to −0.81°/%) fall in the range of the previous reports except for slightly lower HAT magnitude. In the two patients in this study, despite disruption in the HA map, which correlates with the enhancement area in LGE image, elevated MD and decreased FA values are also in agreement with previous findings in myocardial infarction, indicating less restricted diffusivity and degree of diffusion anisotropy because of alterations in microstructure of the tissue.^5^, ^25^, ^31^ In patient 1 slice 4, discontinuity in anterior wall of the HA map resulting from the two proposed MOCO methods compared with MOCO_LRT_ is possibly because of failed MOCO in some DWI frames (Figure S4). An outlier detection approach could be useful to discard outlier image frames with large deformation in such cases to improve the robustness of MOCO in future studies. The difference in the apical HA maps between MOCO_LRT_ and the proposed methods in patient 2 are possibly because of the effects of motion given that a larger amount of motion was observed in apical slices compared with mid and basal slices (Table S1). The effect of extensive motion on the performance of the proposed MOCO methods requires further investigation in larger patient cohorts.

Note that the metrics used in this study to evaluate the effectiveness of MOCO mainly focused on respiratory correction quality by assessing deformation in head to foot direction because this is the main shift direction introduced by respiration. However, myocardial deformation at other angles, although expected to be not as much as in the head to foot direction, was not captured with the current metrics. Further investigation is needed for assessment of image deformation at various angles.

One limitation of the current study is the lack of through‐plane MOCO. However, it has been reported that for short‐axis image orientation, through‐plane motion is minimal compared with long‐axis orientation,^8^, ^11^, ^32^ especially for apical slices because of the nature of the cardiac motion. Future work could either encode the through‐plane direction by performing 3D acquisition or implement 2D slice‐tracking navigators to address the effects of through‐plane motion on diffusion parameter quantification.

Another limitation is that the current study was performed on a scanner equipped with a stronger gradient system (G_max_ = 200 mT/m) than most clinical scanners (G_max_ = ˜40 mT/m). MR systems equipped with ultra‐high performance gradients have opened the door for unprecedented diffusion imaging in the brain^33^ and recently in the heart.^34^ In this study, limited by peripheral nerve stimulation and the nature of the trapezoidal M2 diffusion gradient, actual maximum gradient amplitude and slew rate achieved on scanner for this particular protocol are 120 mT/m and 180 mT/m/ms, respectively. Because of the shorter TE and shorter diffusion block enabled by the stronger gradients, acquired diffusion‐weighted images have relatively high SNR and contrast stability, which is beneficial to the robustness of image registration‐based MOCO algorithm. The feasibility of the proposed MOCO method for data acquired on other systems with lower gradient performance merits further investigation.

The future direction of this work is to implement the MOCO scheme inline along with automatic LV segmentation to enable a fast and streamlined cardiac DTI postprocessing pipeline as well as convenient diffusion maps and quantitative results visualization on the scanner.

## 5 CONCLUSIONS

This study demonstrates a fast and robust MOCO approach using image registration for in vivo free‐breathing cardiac DTI. Between the two proposed MOCO strategies, MOCO_Avg_ was found to be more robust than MOCO_Naive_. The proposed fast MOCO method improves the quality of quantitative diffusion maps and will facilitate clinical translation of cardiac DTI.

## CONFLICT OF INTEREST STATEMENT

The authors Y.L., N.J., P.S., and X.B. are employees of Siemens Healthineers (Erlangen, Germany).

## Supporting information


**Table S1.** Standard deviations of the epicardium pixel position across all image frames in all volunteers. Values are reported as average ± standard deviation. Statistically significant difference (*p* < 0.05) is indicated by the following symbols. Note that all three motion correction (MOCO) methods yield significantly different results compared with free‐breathing, therefore these comparisons are not labeled to make the table clear.
**Table S2.** Average and standard deviation of the global mean diffusivity (MD), fractional anisotropy (FA), and helix angle transmurality (HAT) values in two patients. Note that results of No motion correction (MOCO) are not considered accurate due to motion artifacts.
**Table S3.** Average and standard deviations of mean diffusivity (MD), fractional anisotropy (FA), and helix angle transmurality (HAT) values of each individual slice in patient 2. Note that results of No motion correction (MOCO) are not considered accurate due to motion artifacts.
**Figure S1.** Motion correction (MOCO) strategy flow chart. (A): MOCO_Naive_. (B): MOCO_Avg_. Orange arrows indicate the operation of image registration.
**Figure S2.** Mean diffusivity (MD), fractional anisotropy (FA), and helix angle (HA) maps in one volunteer without motion correction. Note that heterogeneity of MD and FA, and abrupt changes of HA in LV are due to motion artifacts. Therefore, MD, FA and helix angle transmurality (HAT) quantification without motion correction is not reliable.
**Figure S3.** Short‐axis 2D LGE image in patient 1. LGE enhancement indicated by red arrows in the anterior and anterolateral region correlated with the disruption of helical structure shown in Figure 4.
**Figure S4.** (A). Animated individual DWI images resulting from the proposed motion correction (MOCO) methods in all five slices in patient 2. Noticeable large deformation due to failed motion correction was observed in slice #4 resulting from MOCO_Naive_ as indicated by the red box in the figure. (B). An example frame with failed motion correction (deformation is evident in the region indicated by the red arrow) in slice #4 resulting from MOCO_Naive_.
**Figure S5.** (A) Short‐axis 2D LGE images in patient 2 with suspicious sarcoid. (B) Zoomed‐in anteroseptal region of LGE, mean diffusivity (MD), fractional anisotropy (FA), and helix angle (HA) maps in the basal slice. (C) Zoomed‐in anterior and anterolateral region of LGE, MD, FA, and HA maps in the apical slice.
