# Supplementary material for: Toward optimal inline respiratory motion correction for in vivo cardiac diffusion tensor MRI using symmetric and inverse‐consistent deformable image registration
Source: Magn Reson Med. 2025 Mar 10;94(2):724–34. doi: 10.1002/mrm.30485 (PMC12137779; doi:10.1002/mrm.30485)
Supplement: Supplementary file 1 — Table S1. Standard deviations of the epicardium pixel position across all image frames in all volunteers. Values are reported as average ± standard deviation. Statistically significant difference (p < 0.05) is indicated by the following symbols. Note that all three motion correction (MOCO) methods yield significantly different results compared with free‐breathing, therefore these comparisons are not labeled to make the table clear. Table S2. Average and standard deviation of the global mean diffusivity (MD), fractional anisotropy (FA), and helix angle transmurality (HAT) values in two patients. Note that results of No motion correction (MOCO) are not considered accurate due to motion artifacts. Table S3. Average and standard deviations of mean diffusivity (MD), fractional anisotropy (FA), and helix angle transmurality (HAT) values of each individual slice in patient 2. Note that results of No motion correction (MOCO) are not considered accurate due to motion artifacts. Figure S1. Motion correction (MOCO) strategy flow chart. (A): MOCONaive. (B): MOCOAvg. Orange arrows indicate the operation of image registration. Figure S2. Mean diffusivity (MD), fractional anisotropy (FA), and helix angle (HA) maps in one volunteer without motion correction. Note that heterogeneity of MD and FA, and abrupt changes of HA in LV are due to motion artifacts. Therefore, MD, FA and helix angle transmurality (HAT) quantification without motion correction is not reliable. Figure S3. Short‐axis 2D LGE image in patient 1. LGE enhancement indicated by red arrows in the anterior and anterolateral region correlated with the disruption of helical structure shown in Figure 4. Figure S4. (A). Animated individual DWI images resulting from the proposed motion correction (MOCO) methods in all five slices in patient 2. Noticeable large deformation due to failed motion correction was observed in slice #4 resulting from MOCONaive as indicated by the red box in the figure. (B). An example frame with fail [file MRM-94-724-s001.docx]

**Supporting Information**

**Table S1.** Standard deviations of the epicardium pixel position across all image frames in all volunteers. Values are reported as average ± standard deviation. Statistically significant difference (P<0.05) is indicated by the following symbols. Note that all three MOCO methods yield significantly different results compared with free-breathing, therefore these comparisons are not labeled to make the table clear.

*MOCO_LRT_ vs. MOCO_Naive_

†MOCO_LRT_ vs. MOCO_Avg_

|  | Slice #1  (base) | Slice #2 | Slice #3  (mid) | Slice #4 | Slice #5  (apex) | Global |
| --- | --- | --- | --- | --- | --- | --- |
| Free-breathing | 2.33 ± 0.69 | 2.28 ± 0.96 | 2.03 ± 0.83 | 1.88 ± 0.97 | 2.05 ± 0.92 | 2.12 ± 0.80 |
| MOCO_LRT_ | 1.33 ± 0.54 | 1.31 ± 0.52 | 1.15 ± 0.44 | 1.20 ± 0.42 | 1.43 ± 0.67 | 1.28 ± 0.45 |
| MOCO_Naive_ | 0.92 ± 0.48* | 0.81 ± 0.27* | 0.72 ± 0.45* | 0.86 ± 0.36* | 1.04 ± 0.42* | 0.87 ± 0.25* |
| MOCO_Avg_ | 0.92 ± 0.49† | 0.79 ± 0.34† | 0.72 ± 0.48† | 0.87 ± 0.37† | 1.05 ± 0.38† | 0.87 ± 0.27† |

**Table S2.** Average and standard deviation of the global MD, FA, and HAT values in two patients. Note that results of No MOCO are not considered accurate due to motion artifacts.

|  | Global MD (µm^2^/ms) | Global FA  (a.u.) | Global HAT  (°/%) |
| --- | --- | --- | --- |
| Patient 1 | | | |
| No MOCO | 1.56 ± 0.26 | 0.30 ± 0.13 | -0.54 ± 0.36 |
| MOCO_LRT_ | 1.57 ± 0.32 | 0.28 ± 0.13 | -0.63 ± 0.38 |
| MOCO_Naive_ | 1.61 ± 0.20 | 0.25 ± 0.07 | -0.62 ± 0.39 |
| MOCO_Avg_ | 1.63 ± 0.20 | 0.24 ± 0.07 | -0.57 ± 0.42 |
| Patient 2 | | | |
| No MOCO | 1.65 ± 0.36 | 0.30 ± 0.13 | -0.62 ± 0.57 |
| MOCO_LRT_ | 1.61 ± 0.30 | 0.29 ± 0.12 | -0.79 ± 0.42 |
| MOCO_Naive_ | 1.62 ± 0.28 | 0.25 ± 0.08 | -0.66 ± 0.34 |
| MOCO_Avg_ | 1.60 ± 0.23 | 0.25 ± 0.08 | -0.66 ± 0.32 |

**Table S3.** Average and standard deviations of MD, FA, and HAT values of each individual slice in patient 2. Note that results of No MOCO are not considered accurate due to motion artifacts.

|  | Slice #1 (Base) | Slice #2 | Slice #3 | Slice #4 | Slice #5  (Apex) |
| --- | --- | --- | --- | --- | --- |
| MD (µm^2^/ms) | | | | | |
| No MOCO | 1.72 ± 0.43 | 1.57 ± 0.23 | 1.61 ± 0.34 | 1.82 ± 0.44 | 1.56 ± 0.21 |
| MOCO_LRT_ | 1.61 ± 0.25 | 1.60 ± 0.34 | 1.60 ± 0.30 | 1.63 ± 0.28 | 1.61 ± 0.28 |
| MOCO_Naive_ | 1.71 ± 0.33 | 1.56 ± 0.24 | 1.51 ± 0.22 | 1.79 ± 0.28 | 1.56 ± 0.20 |
| MOCO_Avg_ | 1.66 ± 0.23 | 1.55 ± 0.21 | 1.54 ± 0.20 | 1.62 ± 0.22 | 1.60 ± 0.23 |
| FA | | | | | |
| No MOCO | 0.29 ± 0.15 | 0.30 ± 0.09 | 0.30 ± 0.13 | 0.31 ± 0.13 | 0.29 ± 0.12 |
| MOCO_LRT_ | 0.27 ± 0.09 | 0.30 ± 0.11 | 0.28 ± 0.12 | 0.31 ± 0.13 | 0.30 ± 0.13 |
| MOCO_Naive_ | 0.22 ± 0.08 | 0.27 ± 0.08 | 0.26 ± 0.07 | 0.26 ± 0.08 | 0.25 ± 0.10 |
| MOCO_Avg_ | 0.22 ± 0.07 | 0.26 ± 0.07 | 0.26 ± 0.07 | 0.25 ± 0.08 | 0.24 ± 0.10 |
| HAT (°/%) | | | | | |
| No MOCO | -0.77 ± 0.51 | -0.52 ± 0.36 | -0.66 ± 0.29 | -0.50 ± 0.89 | -0.65 ± 0.62 |
| MOCO_LRT_ | -0.60 ± 0.43 | -0.76 ± 0.38 | -0.82 ± 0.32 | -0.93 ± 0.34 | -0.81 ± 0.55 |
| MOCO_Naive_ | -0.73 ± 0.45 | -0.66 ± 0.19 | -0.68 ± 0.21 | -0.55 ± 0.40 | -0.70 ± 0.37 |
| MOCO_Avg_ | -0.70 ± 0.42 | -0.61 ± 0.20 | -0.67 ± 0.19 | -0.68 ± 0.32 | -0.62 ± 0.39 |


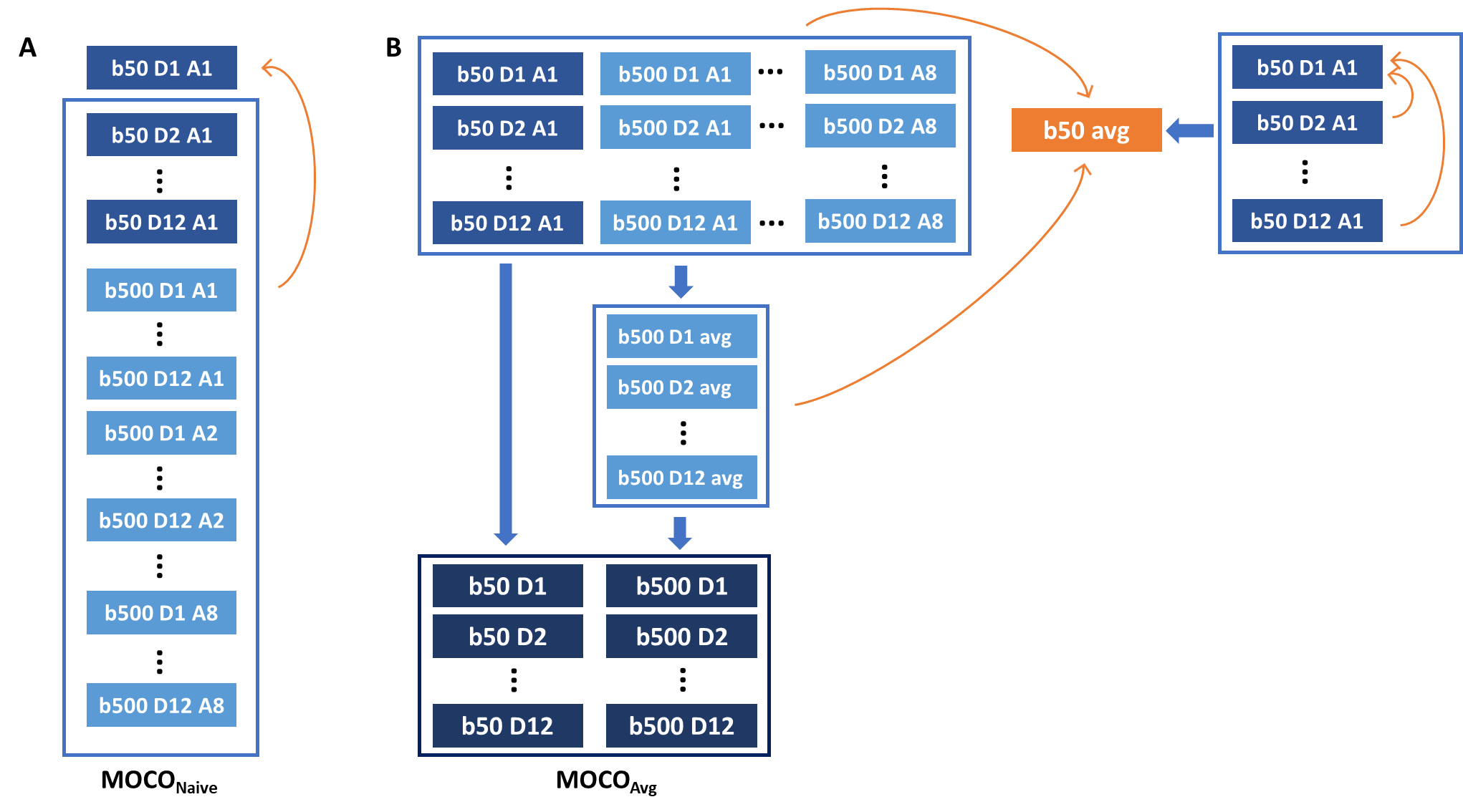


**Figure S1.** MOCO strategy flow chart. A: MOCO_Naive_. B: MOCO_Avg_. Orange arrows indicate the operation of image registration.


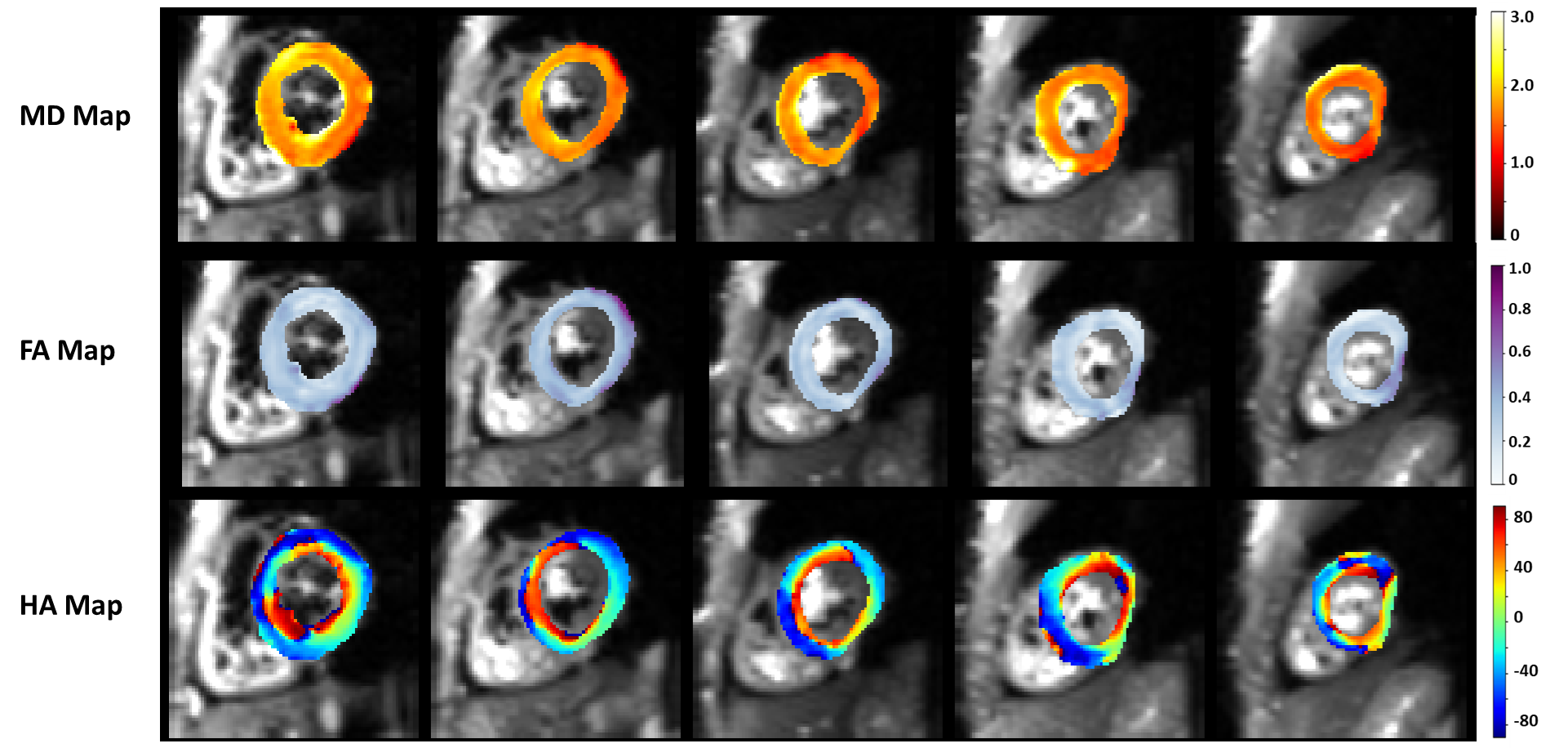


**Figure S2.** MD, FA, and HA maps in one volunteer without motion correction. Note that heterogeneity of MD and FA, and abrupt changes of HA in LV are due to motion artifacts. Therefore, MD, FA and HAT quantification without motion correction is not reliable.


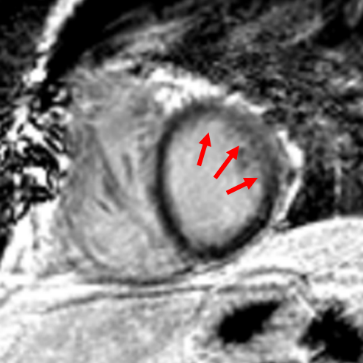


**Figure S3.** Short-axis 2D LGE image in patient 1. LGE enhancement indicated by red arrows in the anterior and anterolateral region correlated with the disruption of helical structure shown in Figure 4.

**
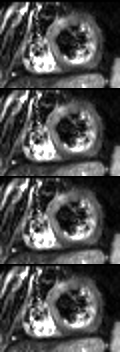

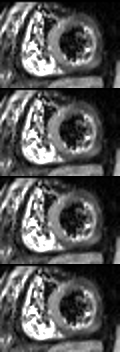

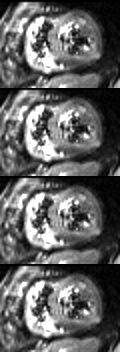

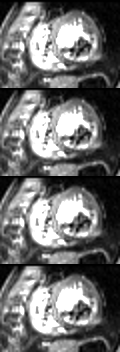

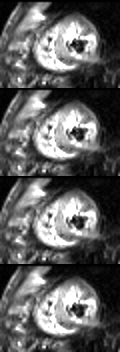
**

**Slice #1**

**MOCO_Naive_**

**MOCO_Avg_**

**Slice #5**

**Slice #4**

**Slice #3**

**Slice #2**

**(A)**

**(B)**

**MOCO_Naive_**

**Slice #4**

**Figure S4.** (A). Animated individual DWI images resulting from the proposed MOCO methods in all five slices in patient 2. Noticeable large deformation due to failed motion correction was observed in slice #4 resulting from MOCO_Naive_ method as indicated by the red box in the figure. (B). An example frame with failed motion correction (deformation is evident in the region indicated by the red arrow) in Slice #4 resulting from MOCO_Naive_.

**
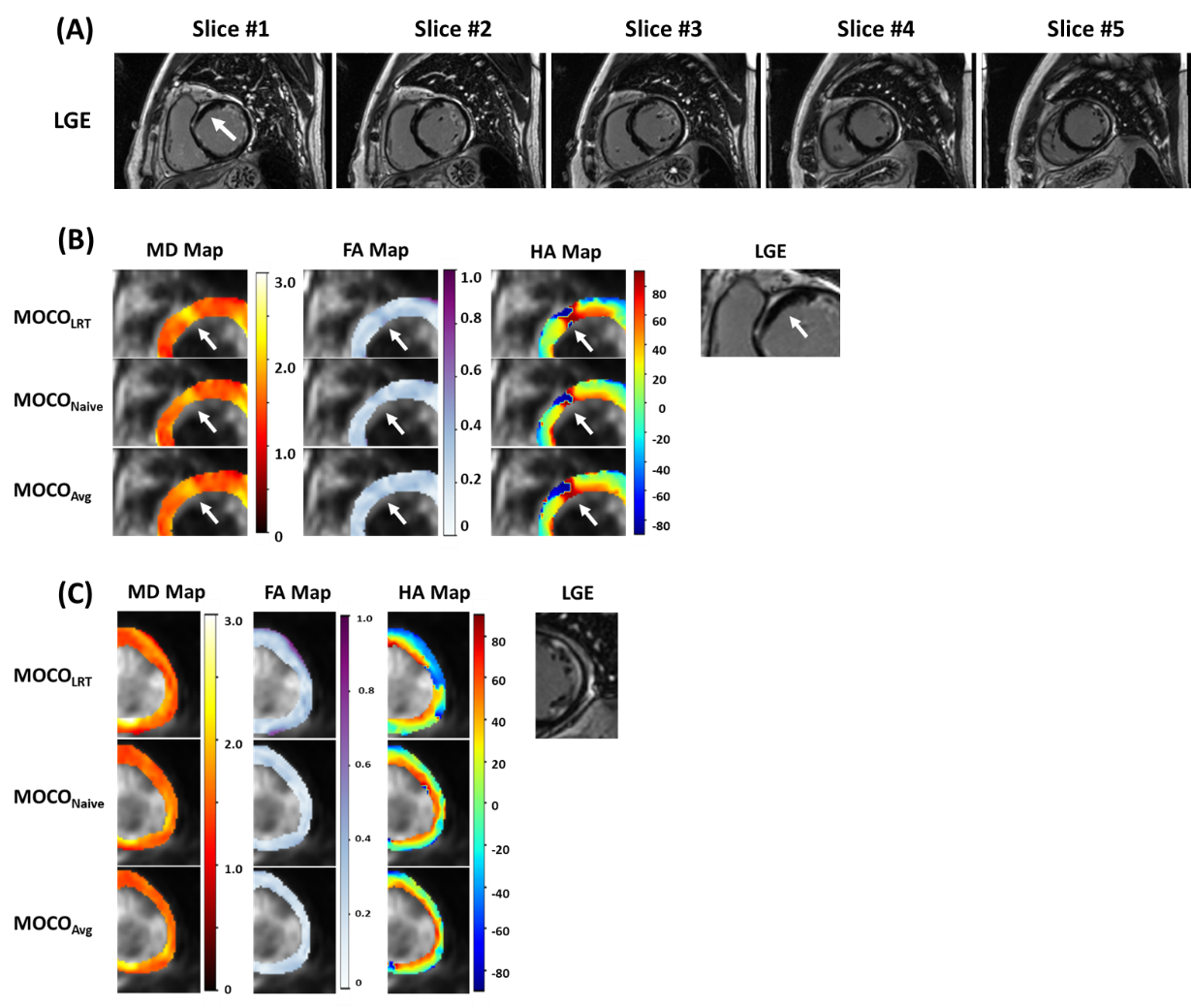
**

**Figure S5. (A)** Short-axis 2D LGE images in patient 2 with suspicious sarcoid. **(B)** Zoomed-in anteroseptal region of LGE, MD, FA, and HA maps in the basal slice. **(C)** Zoomed-in anterior and anterolateral region of LGE, MD, FA, and HA maps in the apical slice.
